# Balance and Reactive Steps in Older Adults With and Without Self-Reported Musculoskeletal Conditions

**DOI:** 10.1093/geroni/igaa057.1683

**Published:** 2020-12-16

**Authors:** Ferdinand Delgado, Cheryl Der Ananian, Daniel Peterson

**Affiliations:** Arizona State University, Phoenix, Arizona, United States

## Abstract

Older adults with musculoskeletal conditions (MSC), including arthritis and osteoporosis, may have a higher risk of falls and falls-related injuries. Differences in balance between individuals with and without self-reported MSC are not well understood. Therefore, this study compared measures of balance (static and dynamic) and reactive stepping between older adults (N=99) with (75.79±5.38 years, n=38, 82% female) and without (75.93±6.36 years, n=61, 67% female) MSC. A cross-sectional design was used. Static balance was assessed via postural sway area (PSA) and PSA root mean square (PSARMS) during quiet stance. Dynamic balance was assessed with the Timed Up & Go (TUG), a dual-task cognitive TUG (TUG-COG), and the Four Square Step Test (FSST). Reactive stepping was measured as the first step latency, length, width, time, total number of recovery steps, and time until balance recovery after a backward lean and release. Linear regression was used to assess group differences. After adjusting for age, sex, body mass index (BMI), and grip strength, there were no significant differences between groups in static balance (PSA (p=0.884); PSARMS (p=0.246)) and reactive stepping outcomes (first step latency (p=0.184); total number of steps (p=0.423); step width (p=0.964)). The other reactive step outcomes are not reported since explained variance was not statistically significant (p>0.05). With dynamic balance, significant group differences showed individuals with MSC took more time to complete TUG (p=0.011) and TUG-COG (p=0.005), but not the FSST (p=0.493). Our findings suggest improving dynamic balance, especially with a walking component, in older adults with self-reported MSC is needed.

